# Microwave-assisted green synthesis of Ag/reduced graphene oxide nanocomposite as a surface-enhanced Raman scattering substrate with high uniformity

**DOI:** 10.1186/1556-276X-9-193

**Published:** 2014-04-28

**Authors:** Kai-Chih Hsu, Dong-Hwang Chen

**Affiliations:** 1 Department of Chemical Engineering, National Cheng Kung University, Tainan 701, Taiwan

**Keywords:** Reduced graphene oxide, Ag nanoparticles, Microwave, Green synthesis, SERS substrate, Uniformity

## Abstract

A nanocomposite of silver nanoparticles/reduced graphene oxide (Ag/rGO) has been fabricated as a surface-enhanced Raman scattering (SERS) substrate owing to the large surface area and two-dimensional nanosheet structure of rGO. A facile and rapid microwave-assisted green route has been used for the formation of Ag nanoparticles and the reduction of graphene oxide simultaneously with L-arginine as the reducing agent. By increasing the cycle number of microwave irradiation from 1 and 4 to 8, the mean diameters of Ag nanoparticles deposited on the surface of rGO increased from 10.3 ± 4.6 and 21.4 ± 10.5 to 41.1 ± 12.6 nm. The SERS performance of Ag/rGO nanocomposite was examined using the common Raman reporter molecule 4-aminothiophenol (4-ATP). It was found that the Raman intensity of 4-ATP could be significantly enhanced by increasing the size and content of silver nanoparticles deposited on rGO. Although the Raman intensities of D-band and G-band of rGO were also enhanced simultaneously by the deposited Ag nanoparticles which limited the further improvement of SERS detection sensitivity, the detectable concentration of 4-ATP with Ag/rGO nanocomposite as the SERS substrate still could be lowered to be 10^−10^ M and the enhancement factor could be increased to 1.27 × 10^10^. Furthermore, it was also achievable to lower the relative standard deviation (RSD) values of the Raman intensities to below 5%. This revealed that the Ag/rGO nanocomposite obtained in this work could be used as a SERS substrate with high sensitivity and homogeneity.

## Background

Surface-enhanced Raman scattering (SERS) has been considered as a powerful analytic technology with wide applications in biomedical sensing, chemical analysis, and environmental monitoring owing to its extremely high sensitivity [1]. The SERS effect can be resulted by the electromagnetic mechanism (EM) and chemical mechanism (CM) [2]. The EM, usually with an enhancement factor (EF) of 10^6^ to 10^8^, arises from the enhanced local electromagnetic field due to the surface plasmon resonance of metal nanostructures which may generate lots of ‘hot spots’ [3,4]. The CM, usually with an EF of 10 to 100, is related to the charge transfer resonances between the probe molecules and the SERS substrates [4-6]. Since EM is the main contributor, the nanoscale characteristics of metallic substrates such as composition, particle size, shape, interparticle gap, fissures, and geometry play important roles in the enhancement of SERS [1,3,7].

The SERS substrates currently developed include metallic rough surfaces, nanoparticle colloids, and periodic nanostructures [1]. Au and Ag nanostructures are the materials mostly used because of their excellent ability to enhance the local electromagnetic field [8,9]. Although some top-down nanopatterning techniques such as lithography can be used for the preparation of SERS substrates with high reproducibility and homogeneity, these techniques are limited by low throughput, high cost, few processable materials, and the difficulty to fabricate the well-controlled nanostructures with efficient and abundant hot spots [1,3]. Thus, most of efforts for the development of SERS substrates have been focused on the synthesis of nanoparticle colloids with specific shapes and the bottom-up fabrication techniques such as the deposition and self-assembly or aggregation of nanoparticle colloids [1,3]. However, it is still a challenge in controlling the size and morphology of nanoparticles and their aggregates, the packing degree of assemblies, and the interparticle gap [1,3,10,11]. Therefore, the fabrication of reliable SERS substrates with high EF and homogeneity remains demanded until now.

On the other hand, graphene, also including graphene oxide (GO) and reduced graphene oxide (rGO), has been used widely in catalysts, supercapacitors, transparent electrodes, electrochemical detection, biomedicine, and so on because of its large specific surface area, high electron mobility, and unique optical, thermal, and mechanical properties [12-19]. Recently, some graphene-based hybrids have also been fabricated for the use in SERS [4,20-24]. These hybrid materials show great potential as SERS substrates because the charge transfer between adsorbed molecules and graphene leads to CM mechanism and the noble metal nanoparticles deposited on graphene result in EM mechanism [4]. Furthermore, it is also expectable that noble metal nanoparticles can be deposited on the two-dimensional plate graphene uniformly due to the flat plane of graphene in nature, leading to the high uniformity of characteristic Raman signal. Ding et al. has reported that the Au/rGO hybrid had good uniformity as a SERS substrate. The relative standard deviation (RSD) of Hg^2+^ Raman signal at 1,618 cm^−1^ was 12.8% [25].

In this work, the fabrication of Ag/rGO nanocomposite as a SERS substrate with high EF and homogeneity was attempted. Ag was chosen because of its lower cost as compared to Au. Furthermore, to achieve the goals of high EF and homogeneity, it was desired to deposit plenty of Ag nanoparticles with uniform size on the substrate. Noteworthily, microwave irradiation which offers rapid and uniform heating of solvents, reagents, and intermediates can provide uniform nucleation and growth conditions [26]. So this technique has been used for the synthesis of many metal nanoparticles [27,28]. Moreover, to reduce or eliminate substances hazardous to human health and the environment, the development of green chemical processes and products is becoming more and more important in the past decade [29,30]. Recently, L-arginine (i.e., one of the most common natural amino acids) has been demonstrated to be useful for the green synthesis of some metal and metal oxide nanoparticles because it not only played a role of reducing agent but also acted as a capping agent [28,31-34]. Accordingly, here, we developed a facile and rapid microwave-assisted green route for the formation of Ag nanoparticles and the reduction of graphene oxide simultaneously using L-arginine as the reducing agent to yield the Ag/rGO nanocomposite. The average size and density of the Ag nanoparticles could be controlled by adjusting the cycle number of microwave irradiation. By the detection of the common Raman reporter molecules, 4-aminothiophenol (4-ATP), the resulting Ag/rGO nanocomposites were demonstrated to be suitable SERS substrates with high sensitivity and outstanding uniformity.

## Methods

Graphite powder (99.9%) was obtained from Bay Carbon, Bay City, MI, USA. Potassium manganite (VII) and sodium nitrate were purchased from J.T. Baker, Phillipsburg, NJ, USA. Sulfuric acid was supplied by Panreac, Barcelona, Spain. Hydrogen peroxide was a product of Showa, Minato-ku, Japan. Sulfuric acid was obtained from Merck, Whitehouse Station, NJ, USA. L-arginine was supplied by Sigma-Aldrich, St. Louis, MO, USA. Silver nitrate was obtained from Alfa Aesar, Ward Hill, MA, USA. 4-Aminothiophenol was the product of Aldrich. All chemicals were of guaranteed or analytical grade reagents commercially available and used without further purification. The water used throughout this work was the reagent grade water produced by a Milli-Q SP ultra-pure-water purification system of Nihon Millipore Ltd., Tokyo, Japan.

GO was prepared from purified natural graphite by a modified Hummers method [35]. Ag/rGO nanocomposite was synthesized by a facile, rapid, and green process according to our previous work on the synthesis of silver/iron oxide nanocomposite [31]. Firstly, 15 mg of graphite oxide was dispersed in 20 mL of deionized water by ultrasonication to form a stable GO colloid solution and then mixed with 10 mL of solution containing AgNO_3_ (300 mM) and L-arginine (60 mg/mL). Next, the solution was transferred into a Teflon beaker and then reduced by different cycles of microwave irradiation (2.45 GHz, 900 W). Each cycle included 50s ‘on’ and 10s ‘off’ for three times. The product was collected by centrifugation and then washed several times with deionized water. The resulting nanocomposites were referred to as 1C, 4C, and 8C according to cycle number of microwave irradiation. Following the above procedures in the absence of AgNO_3_, rGO was prepared to confirm the reduction of GO and for comparison with Ag/rGO nanocomposite.

The particle size and composition were determined by transmission electron microscopy (TEM) and energy-dispersive X-ray (EDX) spectroscopy on a high-resolution field emission transmission electron microscopy (HRTEM, JEOL Model JEM-2100 F, Akishima-shi, Japan). The HRTEM image and selected area electron diffraction (SAED) pattern were obtained by a JEOL Model JEM-2100 F electron microscope at 200 kV. The Ag content of Ag/rGO nanocomposite was also determined by dissolving the sample in a concentrated HCl solution and analyzing the solution composition using a GBC SensAA Dual M/A Series Flame/Furnace atomic absorption spectrometer (AAS). The UV-Vis absorption spectra of the resultant colloid solutions were monitored by a JASCO model V-570 UV/Vis/NIR spectrophotometer, Oklahoma City, OK, USA. The crystalline structures were characterized by X-ray diffraction (XRD) analysis on a Shimadzu model RX-III X-ray diffractometer, Kyoto, Japan, at 40 kV and 30 mA with CuKα radiation (*λ* = 0.1542 nm). Raman scattering was performed on a Thermo Fisher Scientific DXR Raman Microscopy, Waltham, MA, USA, using a 532-nm laser source, and a × 10 objective was used to focus the laser beam onto the sample surface and to collect the Raman signal. The XPS measurements were performed on a Kratos Axis Ultra DLD photoelectron spectrophotometer, Chestnut Ridge, NY, USA, with an achromatic Mg/Al X-ray source at 450 W.

For the study on the SERS property, 0.1 mL of solution containing Ag/rGO nanocomposite (3 mg/mL) was dropped on the glass slide and then dried in a vacuum oven at 35°C to obtain the SERS-active substrate. Next, the SERS-active substrate was immersed in 40 mL of 4-ATP solution for 2 h, then washed with deionized water to remove free molecules and dried in air. Finally, the SERS spectrum of 4-ATP was analyzed by the Thermo Fisher Scientific DXR Raman microscopy using a 532-nm laser source.

## Results and discussion

**Figure 1:**
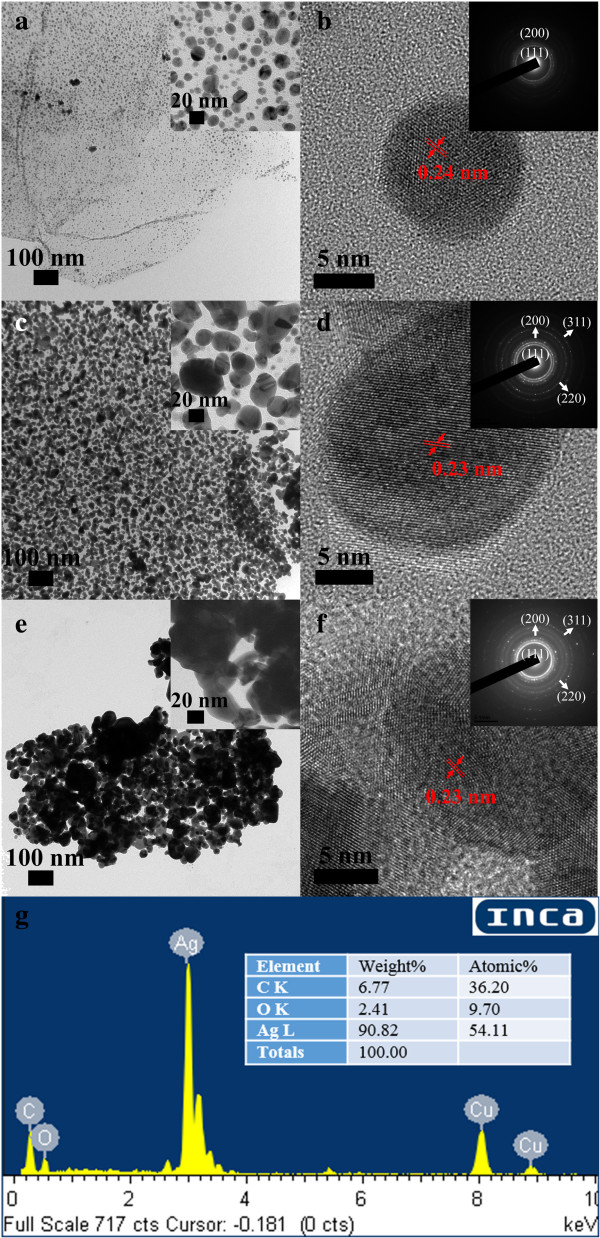
**TEM and HRTEM images of Ag/rGO nanocomposites.** 1C **(a, b)**, 4C **(c, d)**, and 8C **(e, f)**. The insets indicate the SAED patterns. **(g)** The EDX spectrum of Ag/rGO nanocomposite 8C.

Figure 1 shows the TEM and HRTEM images of Ag/rGO nanocomposites 1C, 4C, and 8C. It was found that Ag nanoparticles have been uniformly deposited on rGO successfully. The mean diameters of Ag nanoparticles increased as 10.3 ± 4.6, 21.4 ± 10.5, and 41.1 ± 12.6 nm when the cycle numbers of microwave irradiation were 1, 4, and 8, respectively. The mean diameters of Ag nanoparticles were determined by 300 Ag nanoparticles deposited on rGO. Their HRTEM images all indicated the interlayer spacing of 0.23 to 0.24 nm which related to the (111) plane of face-centered cubic (fcc) Ag. Furthermore, the SAED patterns of Ag/rGO nanocomposites 4C and 8C showed the characteristic rings for the (111), (200), (220), and (311) planes of fcc Ag. For Ag/rGO nanocomposite 1C, the characteristic rings for the (220) and (311) planes of fcc Ag were not significant, probably due to the less Ag content. The EDX analysis of Ag/rGO nanocomposite 8C is indicated in Figure 1g. The presence of Ag confirmed the deposition of Ag nanoparticles. As for the signal of Cu, it was from the copper grid. Furthermore, to confirm the composition, the Ag content of Ag/rGO nanocomposites was also determined by AAS. The weight percentages of Ag in the Ag/rGO nanocomposites 1C, 4C, and 8C were determined to be 37.4%, 69.6%, and 91.6%, respectively. These results revealed that the average size and content of Ag nanoparticles could be controlled by adjusting the cycle number of microwave irradiation.

**Figure 2:**
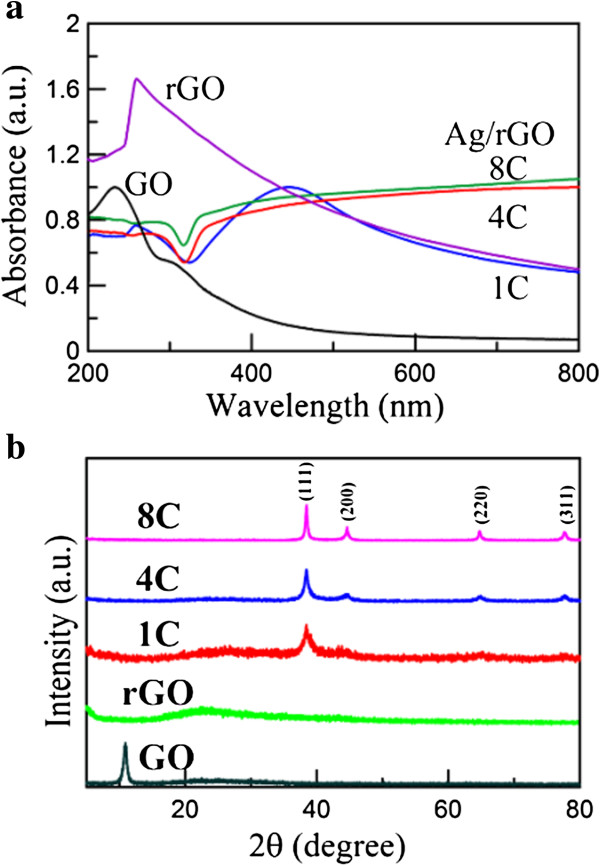
UV-Vis spectra (a) and XRD patterns (b) of GO, rGO, and Ag/rGO nanocomposites 1C, 4C, and 8C.

The UV-Vis absorption spectra of Ag/rGO nanocomposites 1C, 4C, and 8C were shown in Figure 2a, in which the spectra of GO and rGO were also indicated for comparison. The spectrum of GO exhibited the characteristic peaks at 233 and 300 nm, which related to the absorption of C-C and C = O bonds, respectively [36,37]. The characteristic peak of rGO in this work was observed at 260 nm, which was slightly lower than the characteristic peak of highly reduced GO (approximately 268 nm) [36]. This result demonstrated the partial reduction of GO in this work. The successful deposition of Ag nanoparticles on the rGO surface was confirmed by the peaks around 447 nm. With increasing the cycle number of microwave irradiation, the surface plasmon resonance (SPR) bands were redshifted and broadened due to the larger size and aggregation of Ag nanoparticles. This might be due to the substrate effect and the increase in the surface coverage of rGO by Ag nanoparticles [38,39].

The XRD patterns of GO, rGO, and Ag/rGO nanocomposite 1C, 4C, and 8C were shown in Figure 2b. The sharp peak at 2*θ* = 10.56° was due to the (001) plane of GO. However, this peak was not observed in the other XRD patterns, revealing GO has been reduced to rGO. For the XRD patterns of Ag/rGO nanocomposites 4C and 8C, the characteristic peaks at 2*θ* = 38.42°, 44.62°, 64.72°, and 77.68° related to the (111), (200), (220), and (311) planes of fcc Ag, respectively, confirming the formation of Ag nanoparticles on rGO. Nevertheless, for Ag/rGO nanocomposite 1C, only the (111) plane of Ag could be found easily. This might be due to the less Ag content.

**Figure 3:**
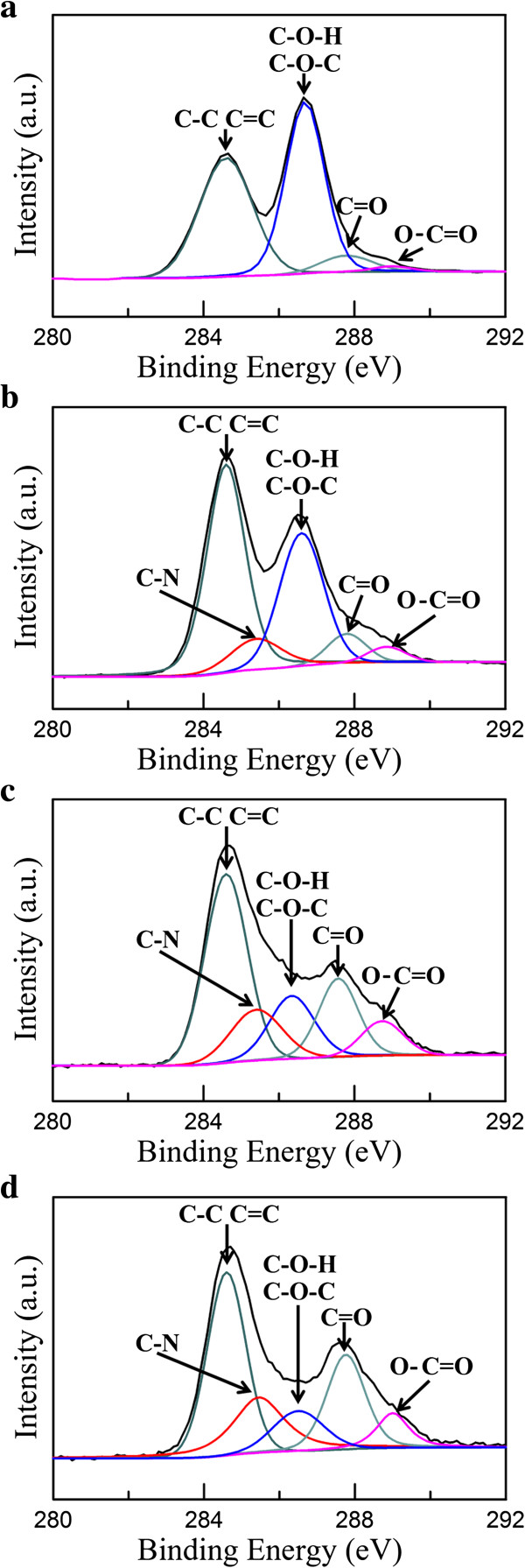
**The C**_**1s **_**XPS spectra of (a) GO and Ag/rGO nanocomposites (b) 1C, (c) 4C, and (d) 8C.**

Figure 3 shows the C1s XPS spectra of GO and Ag/rGO nanocomposites 1C, 4C, and 8C. As illustrated in Figure 3a, the C1s XPS spectrum of GO at 280 to 292 eV showed the characteristic peaks of C-C, C-O-H, C-O-C, C = C, C = O, and O-C = O. They could be attributed to the presence of epoxy, hydroxyl, and carbonyl groups, respectively [36]. From Figure 3b,c,d, with increasing the cycle number of microwave irradiation, the peak intensity of C1s which related to oxygenated functional groups (C-O-H and C-O-C) showed a significant decrease, confirming that most of the epoxide, hydroxyl, and carbonyl functional groups were removed and the degree of reduction of could be enhanced. It was noted that two new characteristic peaks of C-N and O-C = O were observed, and the intensity of C-N and O-C = O could be enhanced with increasing the cycle number of microwave irradiation. This could be reasonably attributed to the increase of arginine capped on the surface of Ag/rGO nanocomposites.

**Figure 4:**
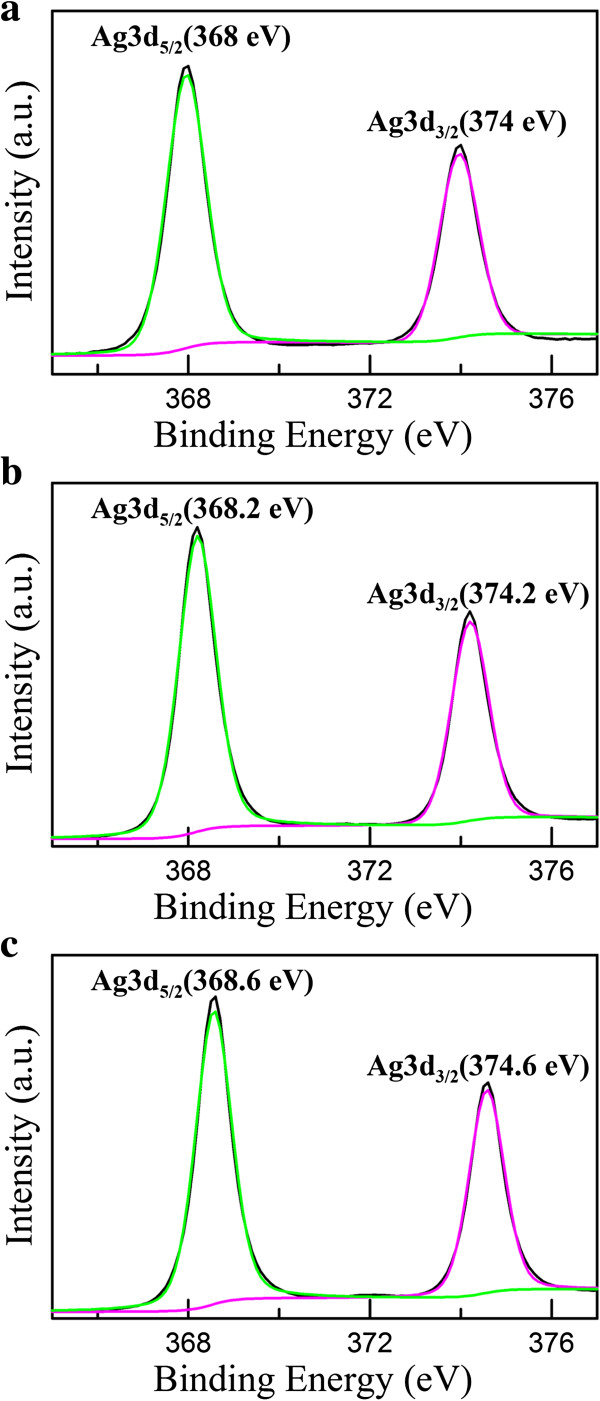
**The Ag**_**3d **_**XPS spectra of Ag/rGO nanocomposites (a) 1C, (b) 4C, and (c) 8C.**

Figure 4 shows the XPS signature of the Ag 3d doublet (3d_5/2_ and 3d_3/2_) for the Ag nanoparticles deposited on rGO. The Ag 3d_5/2_ and 3d_3/2_ peaks of Ag/rGO nanocomposites 1C appeared at 368 and 374 eV, respectively, which shifted to the lower binding energy compared with the characteristic peaks for silver metal at 368.2 and 374.2 eV. In addition, the Ag 3d_5/2_ binding energies have values of 368.2, 367.4, and 367.8 eV for Ag, Ag_2_O, and AgO (with average oxidation states of 0, +1, and +2, respectively) [40]. As a result, slight oxidation on the surface of Ag nanoparticles might be the reason for the negative shift of Ag 3d_3/2_ and Ag 3d_5/2_ binding energy. Moreover, from Figure 4, the binding energy of 3d_3/2_ and Ag 3d_5/2_ increased with increasing the cycle number of microwave irradiation. The results were due to the electron transfer from metallic Ag to the graphene sheets owing to the smaller work function of Ag (4.2 eV) than graphene (4.48 eV) and also proved that the content of Ag nanoparticles could be controlled via adjusting the cycle number of microwave irradiation.

**Figure 5:**
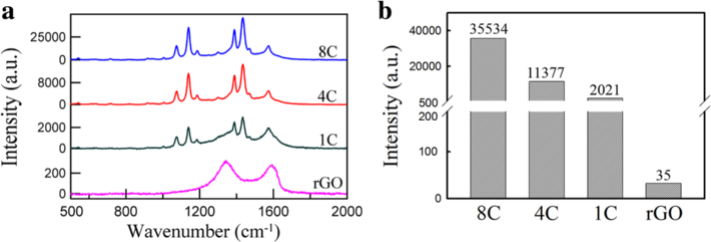
**SERS spectra and intensities. (a)** SERS spectra of 4-ATP at 10^−4^ M on rGO and Ag/rGO nanocomposites 1C, 4C, and 8C. **(b)** SERS intensities of Ag/rGO nanocomposites 1C, 4C, and 8C at 1,140 cm^−1^.

Figure 5a shows the typical SERS spectra of 10^−4^ M 4-ATP acquired from rGO and Ag/rGO nanocomposites 1C, 4C, and 8C. For rGO, only two prominent peaks corresponding to the G and D bands were observed clearly and no evident Raman peaks of 4-ATP could be found. However, for Ag/rGO nanocomposites, the characteristic peaks of 4-ATP were observed clearly. This demonstrated that the Ag/rGO nanocomposites possessed significant SERS property. Their SERS intensities at 1,140 cm^−1^ were indicated in Figure 5b. It was obvious that the peak intensity increased significantly with increasing the cycle number of microwave irradiation. It is known that increasing the number density of Ag nanoparticles on the surface of graphene sheets as hot spots for strong localized EM fields produced by the gap between neighboring Ag nanoparticles [24]. Also, the SERS intensity of Ag nanoparticles usually increased with the increase of particle size. The optimal size of spherical Ag nanoparticles for SERS was about 50 nm [41]. In this work, the mean diameters of Ag nanoparticles increased from 10.3 ± 4.6 to 41.1 ± 12.6 nm when the cycle numbers of microwave irradiation increased from 1 to 8. Thus, the cycle number effect of microwave irradiation could be attributed to the larger size and higher content or number density of Ag nanoparticles.

**Figure 6:**
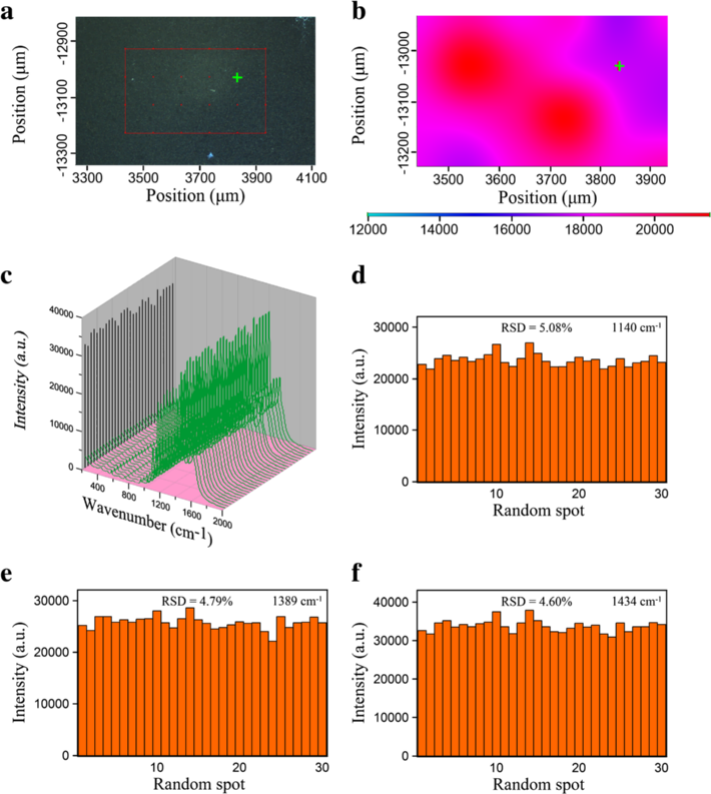
**Optical image, SERS mapping, SERS spectra, and RSD values. (a)** Optical image of an area of 0.5 mm × 0.3 mm for the Ag/rGO nanocomposite 8C substrate. **(b)** The corresponding two-dimensional SERS mapping after 4-ATP adsorption. The peak mapped was at 1,140 cm^−1^. **(c)** A series of SERS spectra randomly collected from 30 spots of the Ag/rGO nanocomposite 8C substrate at 10^−5^ M 4-ATP. **(d to f)** The intensities of three main vibrations at 1,140, 1,389, and 1,434 cm^−1^ in the SERS spectra as shown in **(c)**.

Figure 6a indicates the optical image of an area of 0.5 mm × 0.3 mm for the Ag/rGO nanocomposite 8C substrate. The corresponding two-dimensional SERS mapping (at 1,140 cm^−1^) after 4-ATP adsorption was shown in Figure 6b. It was found that the SERS intensities at different positions had no significant differences. To further investigate the uniformity, a series of SERS spectra randomly collected from 30 spots of the Ag/rGO nanocomposite 8C substrate at 10^−5^ M 4-ATP were shown in Figure 6c. The RSD values of the intensities for three main vibrations at 1,140, 1,389, and 1,434 cm^−1^ were calculated to be 5.08%, 4.79%, and 4.6%, respectively, as indicated in Figure 6d,e,f. Such low RSD values were significantly better than some previous works with lower RSD values and revealed that the resulting Ag/rGO nanocomposite 8C had outstanding uniformity as a SERS substrate [10,11,25]. This could be attributed to the fact that Ag nanoparticles were deposited uniformly on the flat surface of rGO so the closely packed Ag nanoparticles might offer a great deal of uniform hot spots for SERS to enhance the Raman signal of adsorbed molecules. This result revealed that the Ag/rGO nanocomposites could be regarded as an excellent SERS-active substrate with highly uniformity.

**Figure 7:**
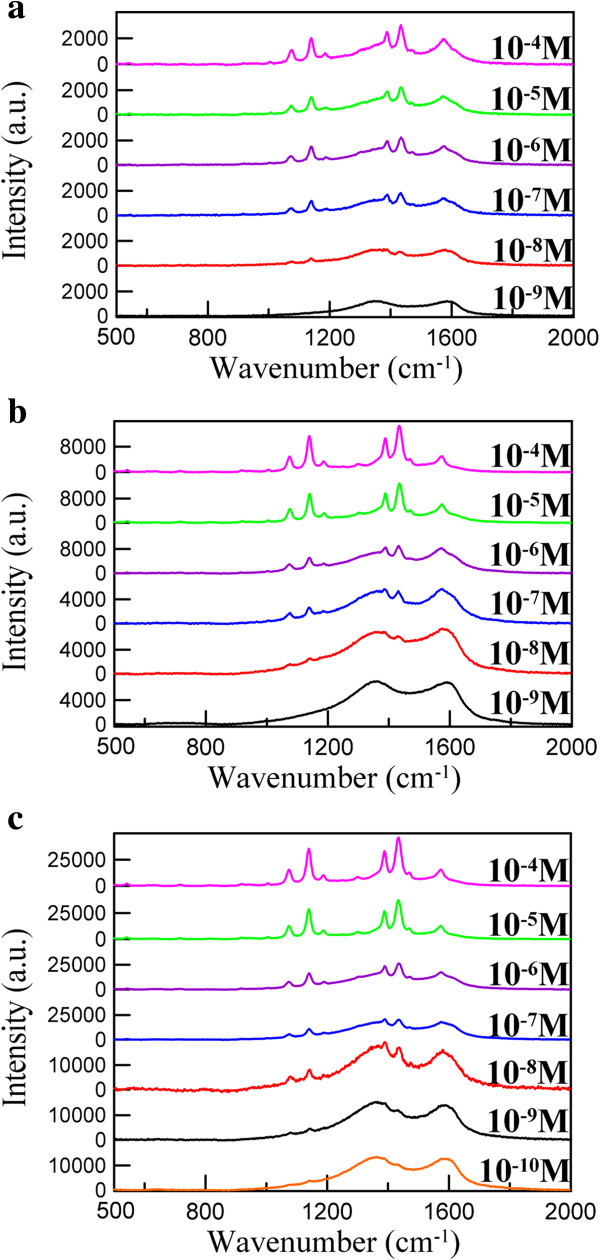
**SERS spectra of 4-ATP on Ag/rGO nanocomposites.** 1C **(a)** and 4C **(b)** at 10^−4^ to 10^−9^ M and 8C **(c)** at 10^−4^ to 10^−10^ M.

Figure 7 shows the SERS spectra of different concentrations of 4-ATP adsorbed on Ag/rGO nanocomposites 1C, 4C, and 8C. The SERS spectrum of 4-ATP on the Ag/rGO nanocomposite exhibited four b_2_ vibration modes at 1,140, 1,389, 1,434, and 1,574 cm^−1^, which could be assigned to *ν*(C-C), *ν*(C-C) + *δ*(C-H), *δ*(C-H) + *ν*(C-C), *δ*(C-H), respectively, and one a_1_ vibration mode of the p,p'-dimercaptoazobenzene molecule at 1,074 cm^−1^ related to *ν*(C-S) [3]. It was obvious that the intensities of the five bands in the SERS spectrum of 4-ATP on the Ag/rGO nanocomposites were enhanced significantly.

The apparent EF of the characteristic Raman signal at 1,140 cm^−1^ in the SERS spectrum of 4-ATP could be estimated according to the following relation [42]:

(1) $$\mathrm{EF}=I_{\mathrm{SERS}}C_{\mathrm{NRS}}/I_{\mathrm{NRS}}C_{\mathrm{SERS}}$$

where *I*_SERS_ and *I*_NRS_ are the SERS intensities on the SERS-active and non-SERS-active substrates, respectively, and *C*_SERS_ and *C*_NRS_ are the corresponding analyte concentrations used. The EF values at 1,140 cm^−1^ for the Ag/rGO nanocomposites 1C and 4C substrates at 10^−8^ M 4-ATP were found to be 1.97 × 10^7^ and 9.04 × 10^7^, respectively. Also, the EF value at 1,140 cm^−1^ for the Ag/rGO nanocomposite 8C substrate at 10^−10^ M 4-ATP was further raised to 1.27 × 10^10^. This demonstrated the EF values for the Ag/rGO nanocomposites could be enhanced by increasing the size and content of Ag nanoparticles on the surface of rGO.

It was mentionable that the closely packed Ag nanoparticles on the surface of rGO not only enhanced the Raman signal of 4-ATP significantly but also enhanced the Raman intensities of D-band and G-band of rGO simultaneously as shown in Figure 7. This limited the further improvement of SERS detection sensitivity. However, in spite of this, the detectable concentration of 4-ATP with the Ag/rGO nanocomposite 8C as the SERS substrate still could be lowered to be about 10^−10^ M and the EF value could be raised to 1.27 × 10^10^. They were better than some previous works [22,42,43]. According to the above results, the Ag/rGO nanocomposite indeed could be used as a SERS substrate with high EF and homogeneity.

## Conclusions

Ag/rGO nanocomposite has been synthesized via a rapid and facile green process. By the use of L-arginine and microwave irradiation, Ag nanoparticles were deposited uniformly on the surface of rGO. The size and content of Ag nanoparticles could be controlled via adjusting the cycle number of microwave irradiation. The Ag/rGO nanocomposite has been demonstrated to be useful as the SERS substrate with high sensitivity and uniformity owing to the uniform deposition of Ag nanoparticles on the flat surface of rGO, offering a lot of hot spots for SERS. Although the Raman intensities of D-band and G-band of rGO were also enhanced and limited the further improvement of SERS detection sensitivity, the detectable concentration of 4-ATP with Ag/rGO nanocomposite as the SERS substrate still could be lowered to be 10^−10^ M and the EF value could be raised to 1.27 × 10^10^. In addition, the RSD values of the intensities could be decreased to below 5%.

## Competing interests

The authors declare that they have no competing interests.

## Authors’ contributions

KCH carried out the experiments and drafted the manuscript. DHC guided the study and modified the manuscript. Both authors read and approved the final manuscript.

## Authors’ information

KCH is currently a PhD student of the National Cheng Kung University (Taiwan). DHC is a distinguished professor of Chemical Engineering Department at National Cheng Kung University (Taiwan).
